# Effect of Macular Vascular Density on Central Visual Function and Macular Structure in Glaucoma Patients

**DOI:** 10.1038/s41598-018-34417-4

**Published:** 2018-10-30

**Authors:** Soo Ji Jeon, Hae-Young Lopilly Park, Chan Kee Park

**Affiliations:** Department of Ophthalmology, Seoul St. Mary’s Hospital, College of Medicine, The Catholic University of Korea, Seoul, Republic of Korea

## Abstract

In patients with glaucomatous parafoveal scotoma, evidence of compromised vascular circulation was commonly seen. The purpose of this study is to evaluate the relationship between macular vascular density (VD) and central visual function and structure in glaucoma patients. We enrolled 46 eyes of normal tension glaucoma (NTG) patients with parafoveal scotoma. All subjects underwent measurement of segmented macular thickness in each layer and optical coherence tomography angiography (OCTA) to assess VD of macula. Correlation coefficients of VD with structural parameters were identified and multivariate regression analyses were performed to verify factors affecting the MD of SITA 10-2. Superficial VD in NFL, GCL and IPL showed significant correlation with thickness of those layers, but deep VD in INL did not show meaningful correlation with any structural parameters. However, deep VD showed significant correlations with central visual field parameters such as MD of SITA 10-2. By multivariate regression analysis, the significant factors affecting central visual function were deep VD. Different multivariate regression models including segmented macular thicknesses were compared and R^2^ value was best for the model with deep VD, not containing superficial VD (R^2^ = 0.326, *p* = 0.001). Assigning subjects as worse or better visual functional group using regression line, deep VD of worse functional group was significantly lower than that of better group. In couclusion, decreased deep VD was an independent risk factor for central scotoma in addition to structural thinning. Taking both macular thickness and vascular circulation into acount, the deterioration of central visual function could be predicted more precisely.

## Introduction

Glaucomatous optic neuropathy often begins as a parafoveal scotoma in normal tension glaucoma^1,2^. Dysfunction of central visual function could cause disability in daily activities like mobility, driving and reading even in the early stage of disease^3,4^.

Evaluating central visual function by standard automated perimetry (SAP) that uses the Swedish interactive threshold algorithm (SITA) 24-2 is difficult, because a structure-function mismatch commonly occurs in patients with glaucomatous parafoveal scotoma. The structure-function mismatch in parafoveal scotoma means the discrepancy between central visual function and structure, and it is well known to be accounted for by the abundance of retinal ganglion cells (RGCs) in the central retina^5^. Although SAP using SITA 10-2 could overcome the RGC richness of the central retina to some degree^6^, we still have had interests if there are other factors that affect the structure-function mismatch in central visual field defect.

We got a suggestion from the point that patients with initial parafoveal scotomas differ from subjects with initial peripheral scotomas in normal tension glaucoma. Systemic factors such as migraine, Raynaud’s phenomenon and hypotension are considered as important risk factors for central scotoma^7–9^. Central scotoma is also correlated with disc hemorrhage^7,8,10^, which is associated with phenomena such as nocturnal blood pressure dip^11^ and nail bed hemorrhage^12^. The common features of these risk factors are vascular incompetence, which leads to the hypothesis that parafoveal scotoma is associated with vascular impairment.

Fluorescein angiography and indocyanine green angiography are methods that directly evaluate retinal and choroidal vasculature. However, those methods are hard to perform under clinical circumstance due to the invasiveness and time-consuming feature. In recent decades, optical coherence tomography (OCT) has been widely used because of noninvasiveness and enhanced visual resolution. OCT angiography (OCTA) has emerged which could visualize retinal and choroidal circulation without invasive injection of dye, and it offers the opportunity to assess those vascular circulation^13^.

The purpose of this study was to evaluate the relationship between macular vasculature and severity of central scotoma as well as macular thickness. Also, we aimed to investigate whether the macular vascular density could influence the structure-function discrepancy in glaucomatous central scotoma.

## Results

**Figure 1:**
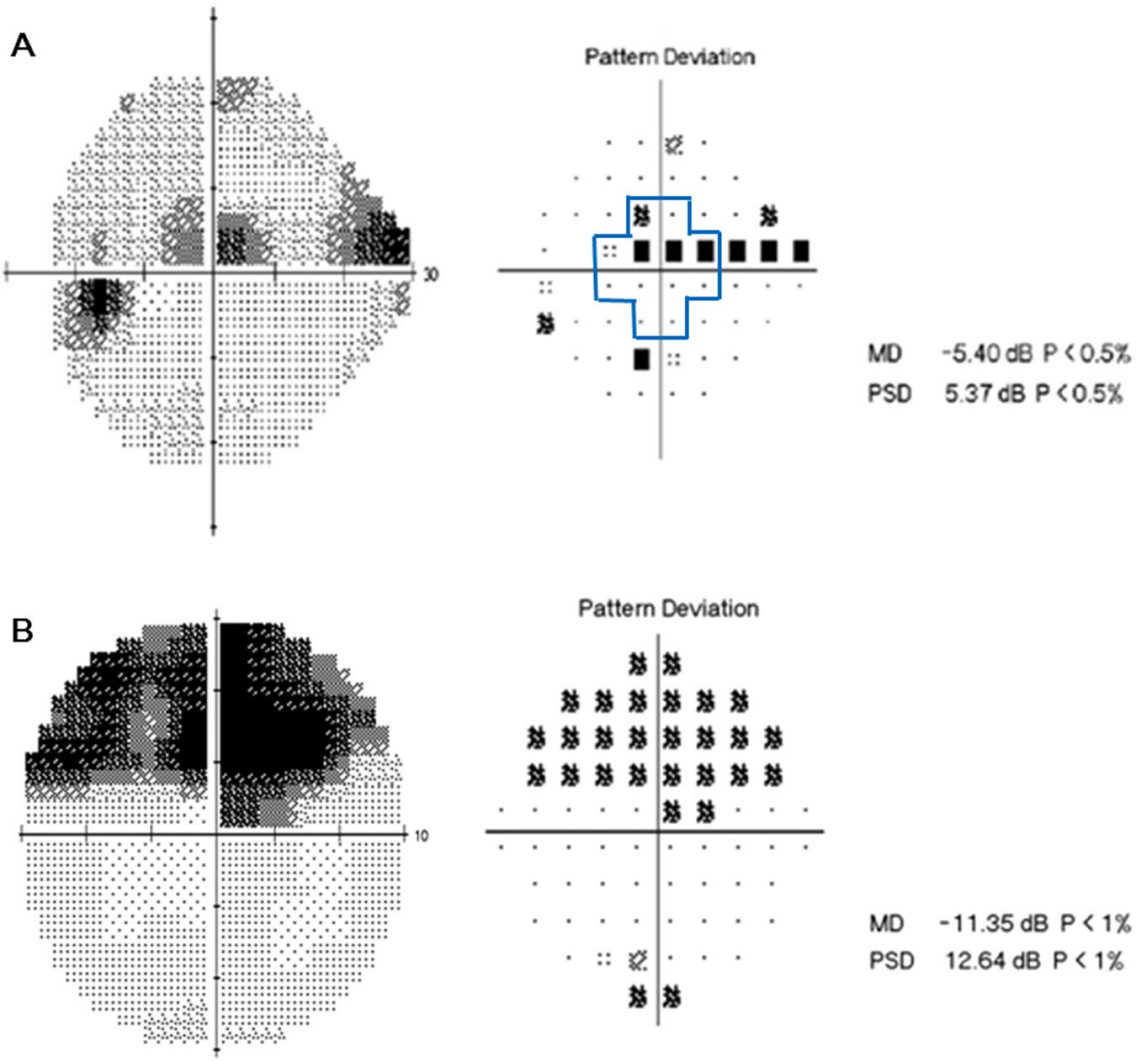
A representative case of a 53-year-old female with central scotoma. (**A**) On pattern deviation probability map of SITA 24-2, clusters of three points with a probability of less than 0.5% were seen within central 10°. (**B**) She performed SITA 10-2 perimetry and central scotoma area was examined minutely.

The baseline characteristics of the 46 subjects are summarized in Table 1. 21 subjects were male and 25 were female, and mean untreated intraocular pressure(IOP) was 16.00 mmHg. The mean axial length was 24.68 ± 1.44 mm and central corneal thickness was 541.16 ± 30.24 μm. The MD and PSD values from SITA 10-2 were worse than the values from SITA 24-2 (−6.37 dB vs. −4.11 dB, respectively, for MD; 7.28 dB vs. 5.78 dB, respectively, for PSD). The correlations between VF sensitivity (1/L) of central 12 points (inside bold lines in Fig. 1) in SITA 24-2, VF sensitivity (1/L) of SITA 10-2 and MD of SITA 10-2 were analyzed to evaluate the repeatability information of VF results, and they all had good correlations (r = 0.828, p < 0.001 for VF sensitivity of 12 points in SITA 24-2 and of SITA 10-2; r = 0.776, p < 0.001 for VF sensitivity of 12 points in SITA 24-2 and MD in SITA 10-2).

**Table 1:** Baseline characteristics of study subjects.

Age, years	59.24 ± 12.28
Gender (Male:Female)	21:25
Intraocular pressure, mmHg	16.00 ± 3.65
Axial length, mm	24.68 ± 1.44
Central corneal thickness, μm	541.16 ± 30.24
MD in SITA 24-2, dB	−4.11 ± 3.43
PSD in SITA 24-2, dB	5.78 ± 3.08
MD in SITA 10-2, dB	−6.37 ± 5.71
PSD in SITA 10-2, dB	7.28 ± 4.40
Best corrected visual acuity, LogMAR	0.03 ± 0.06

Data are presented as means ± standard deviation.

MD = Mean deviation; PSD = Pattern standard deviation.

Several studies have evaluated the quality and repeatability of the density of retinal vascular plexus by OCTA^14–17^. Intraclass correlation coefficients (ICCs) of our VD measurement were 0.799 for superficial VD and 0.846 for deep VD, both showed good to excellent repeatabilities (all *p* < 0.05).

To investigate the trends in structural and vascular circulatory changes according to central visual function, subjects were divided into two groups on the basis of MD in SITA 10-2 (Table 2). In group of better MD in SITA 10-2, the functional parameters(MD and PSD) in SITA 24-2, average cpRNFL and macular ganglion cell-inner plexiform layer (GCIPL) thickness were better than those of worse MD group as expected (*p* < 0.001 for functional parameters; *p* = 0.002 for cpRNFL; 0.012 for GCIPL). Segmented thickness and volume of NFL, GCL and IPL were thinner and smaller in worse MD group (*p* = 0.038, 0.002 and 0.007 for thickness, respectively; *p* = 0.047, 0.001 and 0.007 for volume, respectively). The mean values of macular vascular densities were different between two groups, but only deep vascular layer showed statistical significance (28.85% vs. 27.73%, *p* = 0.212 for superficial VD; 32.11% vs. 31.03%, *p* = 0.037 for deep VD).

**Table 2:** Baseline Characteristics of Subjects according to Central Visual Field Defect Severity based on SITA 10-2 MD value.

	Early (MD ≥ −6dB) (N = 24)	Mod~severe (MD < −6dB) (N = 22)	*P* Value
Age (years)	57.33 (±13.27)	61.95 (±10.48)	0.214
Axial length (cm)	24.77 (±1.63)	24.59 (±1.28)	0.714
**SITA 24-2**			
MD (dB)	−2.01 (±2.09)	−6.21 (±3.24)	<0.001
PSD (dB)	4.02 (±2.19)	7.55 (±2.85)	<0.001
Average cpRNFL thickness (μm)	75.39 (±8.06)	66.91 (±9.25)	0.002
mGCIPL thickness (μm)	70.39 (±7.56)	64.32 (±8.05)	0.012
**Macular segmentation**			
NFL average thickness (μm)	25.17 (±3.41)	22.87 (±3.77)	0.038
NFL volume (mm^3^)	0.76 (±0.11)	0.69 (±0.13)	0.047
GCL average thickness (μm)	35.94 (±4.36)	30.42 (±6.06)	0.002
GCL volume (mm^3^)	0.92 (±0.09)	0.79 (±0.12)	0.001
IPL average thickness (μm)	31.01 (±2.77)	28.28 (±3.71)	0.007
IPL volume (mm^3^)	0.79 (±0.07)	0.74 (±0.08)	0.030
INL average thickness (μm)	36.69 (±2.35)	37.82 (±3.15)	0.169
INL volume (mm^3^)	0.97 (±0.06)	0.98 (±0.08)	0.666
OPL average thickness (μm)	30.22 (±2.68)	32.08 (±4.94)	0.148
OPL volume (mm^3^)	0.80 (±0.06)	0.85 (±0.11)	0.128
ONL average thickness (μm)	61.63 (±5.83)	62.09 (±10.07)	0.860
ONL volume (mm^3^)	1.65 (±0.15)	1.66 (±0.25)	0.971
**Macular vascular density (VD)**			
Supf VD (%)	28.85 (±3.38)	27.73 (±2.56)	0.212
Deep VD (%)	32.11 (±1.39)	31.03 (±2.27)	0.037

Student t-tests was used.

MD = Mean deviation; PSD = Pattern standard deviation; cpRNFL = Circumpapillary retinal nerve fiber layer; mGCIPL = Macular ganglion cell-inner plexiform layer; NFL = Nerve fiber layer; GCL = Ganglion cell layer; IPL = Inner plexiform layer; INL = Inner nuclear layer; OPL = Outer plexiform layer; ONL = Outer nuclear layer; VD = Vascular density.

**Figure 2:**
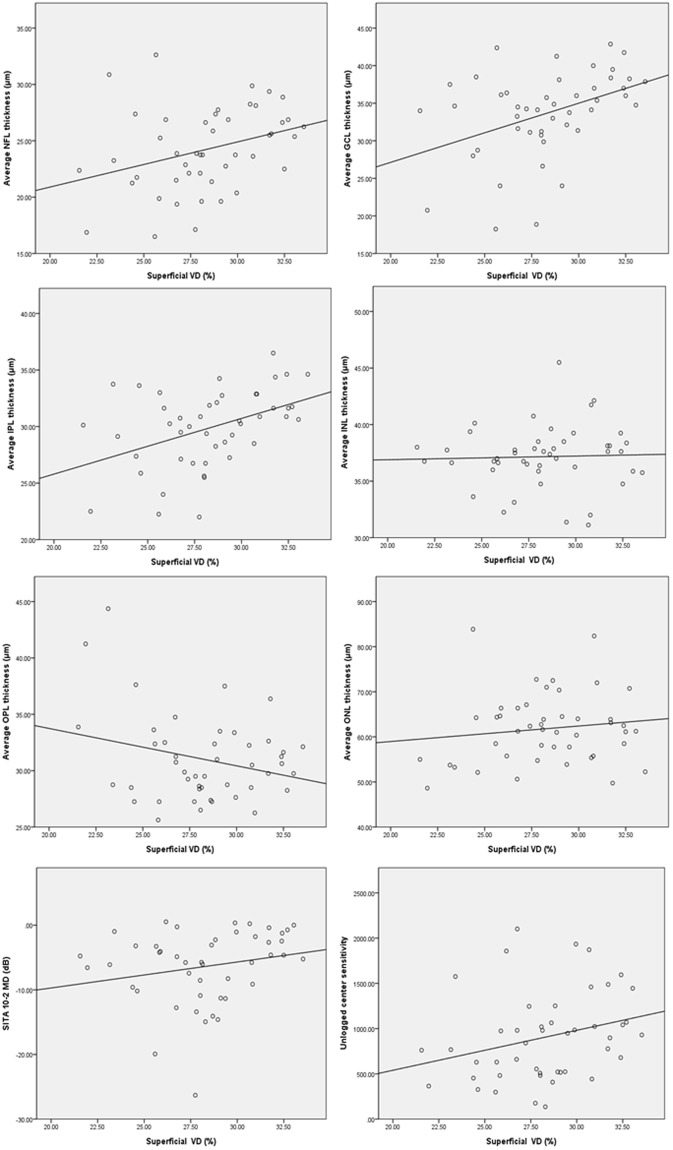
Correlations between superficial macular VD and macular structures or central visual function are shown in scatter plots. Superficial VD versus macular average NFL, GCL, IPL, INL, OPL and ONL thicknesses, MD of SITA 10-2 and unlogged center sensitivity of SITA 24-2. Pearson correlation analysis was used.

**Figure 3:**
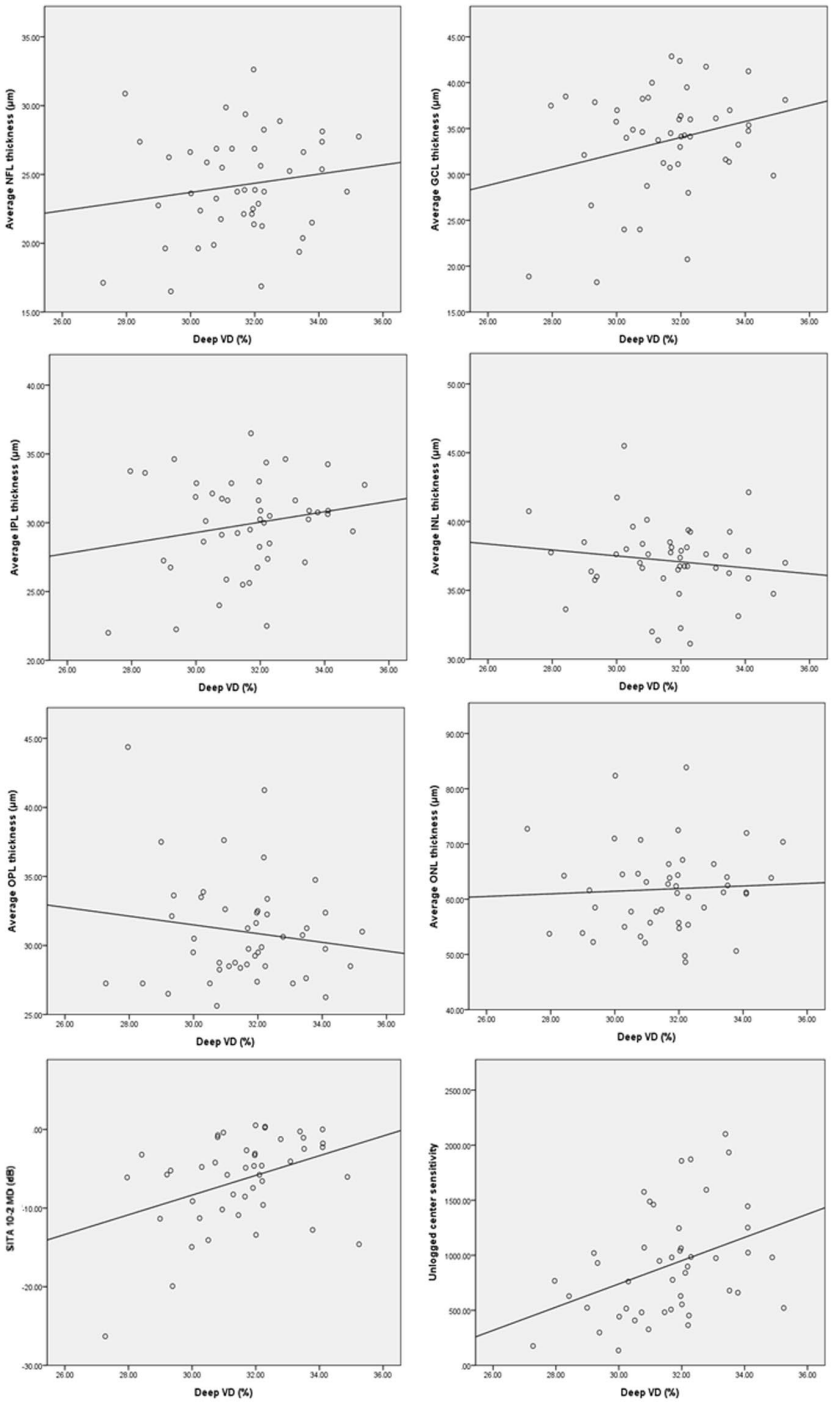
Correlations between deep macular VD and macular structures or central visual function are shown in scatter plots. Deep VD versus macular average NFL, GCL, IPL, INL, OPL and ONL thicknesses, MD of SITA 10-2 and unlogged center sensitivity of SITA 24-2. Pearson correlation analysis was used.

Table 3 showed the correlations between vascular densities and various structural or functional parameters. Superficial VD showed significant correlation with GCIPL and segmented NFL, GCL, IPL thickness, but deep VD did not show meaningful correlation with any structural parameters. In Figs 2 and 3, superficial VD increased according to thickness of NFL, GCL and IPL, but this tendencies were attenuated in deep VD, and there was not significant relationship between deep VD and INL in which we measured the density of deep vascular layer.

**Table 3:** Correlation coefficients between vascular density and structural or functional parameters.

	Superficial VD		Deep VD	
	Correlation coefficient	*P* value	Correlation coefficient	*P* value
Average cpRNFL thickness (μm)	0.278	0.062	0.288	0.052
mGCIPL thickness (μm)	0.476	0.001	0.233	0.123
**Macular segmentation**				
NFL average thickness (μm)	0.326	0.027	0.159	0.290
NFL volume (mm^3^)	0.362	0.013	0.180	0.230
GCL average thickness (μm)	0.412	0.004	0.269	0.071
GCL volume (mm^3^)	0.428	0.003	0.291	0.059
IPL average thickness (μm)	0.433	0.003	0.196	0.192
IPL volume (mm^3^)	0.421	0.004	0.168	0.264
INL average thickness (μm)	0.036	0.814	−0.141	0.351
INL volume (mm^3^)	0.067	0.656	−0.131	0.386
OPL average thickness (μm)	−0.260	0.080	−0.146	0.332
OPL volume (mm^3^)	−0.235	0.115	−0.142	0.348
ONL average thickness (μm)	0.134	0.375	0.054	0.720
ONL volume (mm^3^)	0.135	0.373	0.059	0.696
**SITA 24-2**				
MD (dB)	0.297	0.045	0.279	0.060
PSD (dB)	−0.280	0.060	−0.156	0.300
Unlogged center sensitivity	0.273	0.066	0.385	0.008
**SITA 10-2**				
MD (dB)	0.212	0.156	0.390	0.007
PSD (dB)	−0.311	0.035	−0.187	0.215

Pearson correlation analysis was used.

VD = Vascular density; cpRNFL = Circumpapillary retinal nerve fiber layer; mGCIPL = Macular ganglion cell-inner plexiform layer; NFL = Nerve fiber layer; GCL = Ganglion cell layer; IPL = Inner plexiform layer; INL = Inner nuclear layer; OPL = Outer plexiform layer; ONL = Outer nuclear layer; MD = Mean deviation; PSD = Pattern standard deviation.

The noticeable results in Table 3 were the relationships between VD and VF test. Deep VD showed significant correlation with unlogged center sensitivity (mean value of sensitivity within central 10° as shown in Fig. 1) of SITA 24-2 as well as MD of SITA 10-2 (*r* = 0.385 and 0.390; *p* = 0.008 and 0.007, respectively). However, superficial VD showed different patterns – correlated with MD of SITA 24-2 and PSD of SITA 10–2 *(r* = 0.297 and −0.311; *p* = 0.045 and 0.035, respectively).

**Figure 4:**
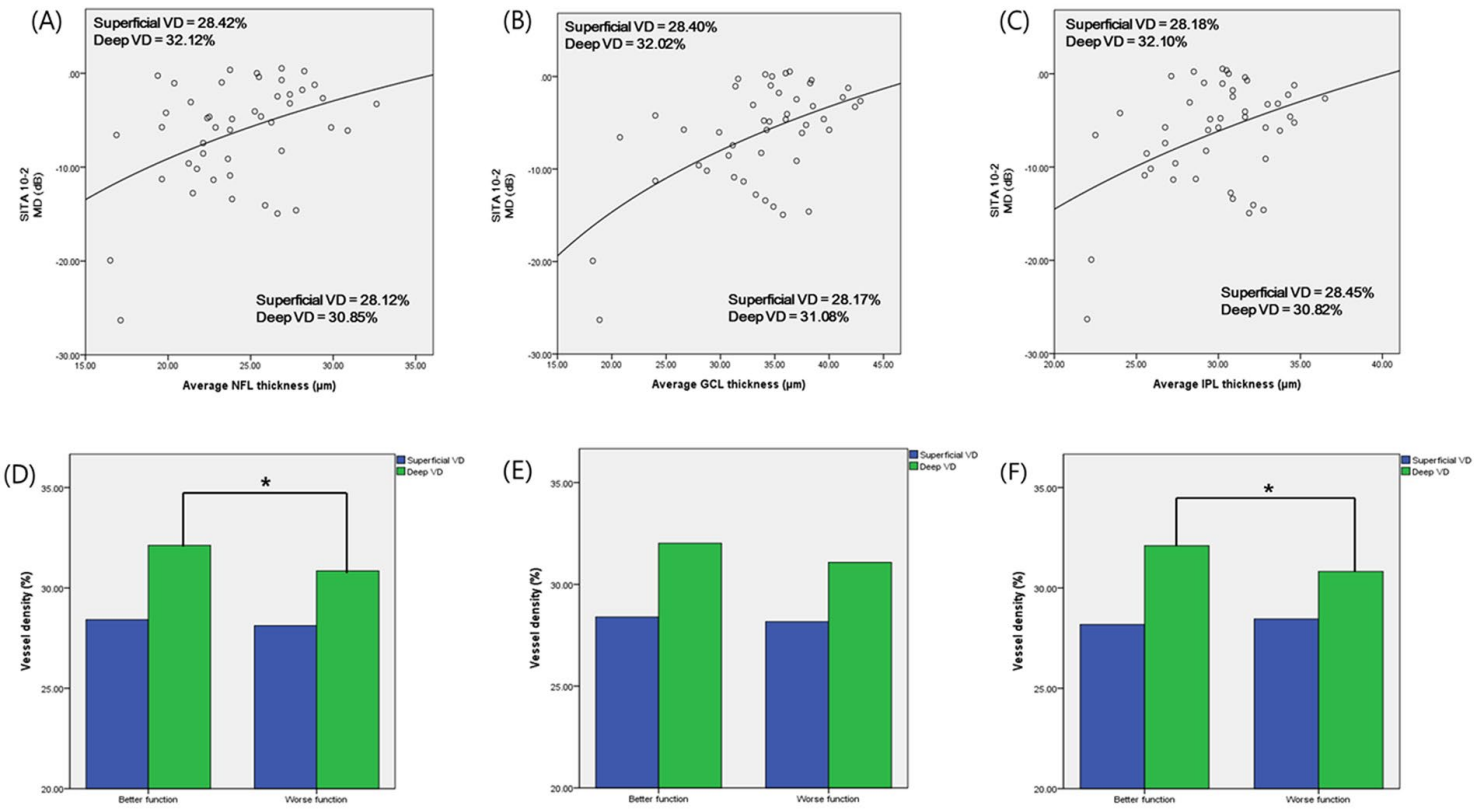
After dividing subjects into two groups based on logarithmic regression line showing the relationship between inner retinal segmented thicknesses and functional parameters (MD of SITA 10-2), mean VD were compared by Student t-test. (These graphs included only NFL, GCL and IPL thickness because those layers only have meaningful correlations with MD of SITA 10-2). *p value less than 0.05.

Figure 4 showed the different mean VD values after dividing subjects into two groups based on the logarithmic regression line showing the relationship between structural parameters and functional parameters (MD of SITA 10-2). The patients with relatively worse function than expected (MD values below the regression line) had lower VD (except the case of superficial VD in IPL-MD graph). When patients were divided based on the regression line showing the relationship between MD and NFL, VD of patients with better function was 28.42% in superficial layer and 32.12% in deep layer, versus patients with worse function was 28.12% in superficial layer and 30.85% in deep layer. The results were similar for regression graphs showing the relationships between MD and GCL or IPL (Fig. 4A–C). However, only the *p*-values for deep VD that compared patients with better and worse function were statistically significant in NFL and IPL as follows; *p* = 0.014 and 0.015 – not significant in superficial VD (Fig. 4D,F). The deep VD difference between functional groups in GCL-MD graph had marginal statistical value (*p* = 0.074).

Univariate and multivariate regression analysis were performed for determining which factors have effect on central visual function. Systemic conditions including hypertension, cardiovascular disease, migraine and Raynaud’s phenomenon were not significant factors for central visual function (all *p* > 0.2). As shown in Table 4, multivariate regression analysis identified GCL average thickness as the significant factor affecting MD from SITA 10-2 (*p* = 0.009 in model 1), and deep VD as marginally significant factor (*p* = 0.090 in model 1). After eliminating factors which were not statistically significant step-by-step, deep VD as well as GCL thickness turned out to be statistically meaningful (*p* = 0.044 for deep VD; *p* = 001 for GCL thickness in model 2). Similarly, in Table 5, deep VD and GCL thickness were marginally significant factors affecting center sensitivity from SITA 24-2 (*p* = 0.066 and 0.077). After eliminating factors which were not statistically significant step-by-step, deep VD was still statistically meaningful factor (*p* = 0.031 in model 2).

**Table 4:** Univariate and multivariate regression analysis of MD of SITA 10-2.

	Univariate analysis		Multivariate analysis Model 1		Multivariate analysis Model 2	
	β ± SE	*P* value	β ± SE	*P* value	β ± SE	*P* value
Superficial VD	0.402 ± 0.279	0.156				
Deep VD	1.253 ± 0.446	0.007	0.720 ± 0.415	0.090	0.851 ± 0.409	0.044
NFL average thickness	0.612 ± 0.215	0.006	−0.342 ± 0.387	0.383		
GCL average thickness	0.533 ± 0.126	<0.001	1.046 ± 0.382	0.009	0.461 ± 0.126	0.001
IPL average thickness	0.687 ± 0.229	0.004	−0.703 ± 0.509	0.175		
INL average thickness	−0.488 ± 0.307	0.119				
OPL average thickness	−0.445 ± 0.224	0.843				
ONL average thickness	−0.082 ± 0.110	0.461				

Model 1 included the factors of p value lower than 0.1 in univariate analysis.

Modes 2 used the backward elimination method.

cpRNFL = Circumpapillary retinal nerve fiber layer; mGCIPL = Macular ganglion cell-inner plexiform layer; VD = Vascular density; NFL = Nerve fiber layer; GCL = Ganglion cell layer; IPL = Inner plexiform layer; INL = Inner nuclear layer, OPL = Outer plexiform layer; ONL = Outer nuclear layer.

**Table 5:** Univariate and multivariate regression analysis of unlogged center sensitivity of SITA 24-2.

	Univariate analysis		Multivariate analysis Model 1		Multivariate analysis Model 2	
	β ± SE	*P* value	β ± SE	*P* value	β ± SE	*P* value
Superficial VD	44.201 ± 23.458	0.066	23.542 ± 24.493	0.342		
Deep VD	105.595 ± 38.201	0.008	74.089 ± 39.195	0.066	85.636 ± 38.491	0.031
NFL average thickness	28.791 ± 19.376	0.144				
GCL average thickness	30.001 ± 11.938	0.016	53.000 ± 29.168	0.077	22.896 ± 11.874	0.060
IPL average thickness	35.277 ± 20.795	0.097	−62.830 ± 48.901	0.206		
INL average thickness	−73.836 ± 24.495	0.103				
OPL average thickness	−16.944 ± 18.982	0.377				
ONL average thickness	−11.326 ± 9.333	0.231				

Model 1 included the factors of p value lower than 0.1 in univariate analysis.

Modes 2 used the backward elimination method.

cpRNFL = Circumpapillary retinal nerve fiber layer; mGCIPL = Macular ganglion cell-inner plexiform layer; VD = Vascular density; NFL = Nerve fiber layer; GCL = Ganglion cell layer; IPL = Inner plexiform layer; INL = Inner nuclear layer, OPL = Outer plexiform layer; ONL = Outer nuclear layer.

The results of various regression analysis models including macular vascular density and inner segmented macular thickness as variables were compared in Table 6. The difference of four analysis models were existence of macular VD in variables. The adjusted R^2^ was best in model 3 and model 4 follows, which included segmented macular thicknesses and deep VD as variables. Model 2 which included only superficial VD and structural parameters showed a lower R^2^ value than model 3 and 4 which included deep VD (adjusted R^2^ = 0.281 for model 2, 0.326 for model 3 and 0.309 for model 4, all *p* < 0.05). R^2^ differences between models were calculated; consistently with the above results, the difference between model 1 and 3 was greatest (R square change = 0.041, *p* = 0.107).

**Table 6:** Adjusted R^2^ of multivariate regression analysis in MD of SITA 10-2.

	Variables in the model	β ± SE	Adjusted R^2^	*P* value
Model 1	NFL average thickness	−0.502 ± 0.396	0.298	0.001
	GCL average thickness	1.168 ± 0.383		
	IPL average thickness	−0.695 ± 0.536		
	INL average thickness	−0.248 ± 0.278		
Model 2	Superficial VD	0.040 ± 0.267	0.281	0.002
	NFL average thickness	−0.498 ± 0.403		
	GCL average thickness	1.164 ± 0.391		
	IPL average thickness	−0.704 ± 0.548		
	INL average thickness	−0.252 ± 0.283		
Model 3	Deep VD	0.691 ± 0.419	0.326	0.001
	NFL average thickness	−0.390 ± 0.394		
	GCL average thickness	1.104 ± 0.388		
	IPL average thickness	−0.605 ± 0.528		
	INL average thickness	−0.206 ± 0.274		
Model 4	Superficial VD	−0.026 ± 0.265	0.309	0.002
	Deep VD	0.697 ± 0.429		
	NFL average thickness	−0.392 ± 0.401		
	GCL average thickness	1.007 ± 0.394		
	IPL average thickness	−0.596 ± 0.540		
	INL average thickness	−0.204 ± 0.279		

cpRNFL = Circumpapillary retinal nerve fiber layer; NFL = Nerve fiber layer; GCL = Ganglion cell layer; IPL = Inner plexiform layer; INL = Inner nuclear layer; VD = Vascular density.

## Discussion

The most important results of various studies of patients with NTG indicate that those with a central VF defect show problems with the vascular component^7–12^. Several studies have shown direct evidence of compromised vascular circulation in those patients. Yoo *et al*. reported smaller retinal arteriolar diameter in NTG patients with parafoveal scotoma^18^. Recently, foveal avascular zone (FAZ) have been demonstrated as the factor affecting visual function in glaucoma using OCTA^19,20^. Kwon *et al*. demonstrated that patients with a central visual defect had an enlarged FAZ and that the area of FAZ was significantly related to the severity of central scotoma^21^. Recently, Penteado *et al*. suggested the superifcial macular VD as a good functional parameter of central visual field^22^. These findings could support and visualize microvascular incompetence in glaucoma patients with central scotoma. Our study also focused on changes in the vascular status of the macula in patients who had early central scotoma with a glaucomatous optic disc, not only for superficial layer but deep layer.

As shown in Table 3, superficial macular VD was correlated with structural and functional measurements, and deep macular VD did not show significant correlations with any structural parameter. However, deep macular VD was related to the parameters representing central visual function such as MD of SITA 10-2 and central sensitivity from SITA 24-2. By multivariate regression analysis, deep macular VD had a role on determining functional parameters along with structural parameters (Tables 4, 5). The evaluation of different models assessing the effect of variables on MD from SITA 10-2 revealed that the power of explanation represented by adjusted R^2^ values was stronger with deep VD plus structural parameters than with superficial VD or with only structural indices. These outcomes imply an independent effect for deep macular VD on changes in the visual fields of our study participants.

Our results showed denser retinal capillary of deep layer in subjects who were functionally better than those of functionally worse subjects (Fig. 4). In scatter plots of those comparisons, one outlier was found and that point might be the result of floor effect of macular structure. However, even when we removed that subjects, grouping according to logarithmic regression line were constant in NFL thickness-MD graph. In GCL-MD and IPL-MD graphs, the trends of comparison were maintained and statistical significance also remained constant (*p* = 0.084 for GCL, 0.032 for IPL).

Several other studies were performed similarly to evaluate the relationship between visual function and VD. Takusagawa *et al*. suggested that only superficial VD was correlated with VF sensitivity^23^. Another study proposed that superficial VD was related with only mGCIPL and deep VD did not show significant relationship with any factor^24^. These findings were in conflict with our results. The main difference between those results and ours are the different study subjects. NTG subjects with central scotoma were only included in our study which were known to have a prominent relationship with vascular incompetence. Superficial retinal vascular layer may have effect on visual field deficits, but the influence could be confused because of the location of the superficial vascular network. It is difficult to determine whether decreased superficial VD of our subjects was independent factor of central scotoma or secondary epiphenomenone due to thinning of the NFL or GCL. However, the deep retinal vessels did not show meaningful relationships with structural parameters, even with INL, the layer where deep VD was measured. So the meaningful correlations between deep VD and central VF parameters might be explained by the independent effect of deep retinal circulation on central visual function seperately from retinal structural thinning. In other words, the deep macular vasculature (which are located in INL) could not be affected by thinning of RNFL and GCL, so deep VD may be more reliable surrogator representing systemic vascular incompetence. Considering this point, it would make sense that deep VD was lower in NTG patients with central scotoma.

Distinctive deep retinal vascular phenomenon could be additionally described through vasoconstriction in the deep retinal capillary under ischemic condition. Many previous studies have presented evidence that might support this hypothesis about hypoxic conditions such as diabetes. Chen *et al*. studied the retina of diabetic patients without retinopathy, and reported that vascular circulation was decreased in the deep retinal layer but not in the superficial layer^25^. More histological changes were found in deep capillary layer of diabetic patients than in the superficial capillary layer^26,27^. A study of diabetic mice found decreases in the retinal capillaries only located in the deep retinal layer^28^. Glaucoma patients with central scotoma also have features of impaired systemic vascular circulation, accordingly, the effect of decreased circulation may be more prominent in deep retinal capillary layer.

Several studies also demonstrated that there were decreased VD in both nonarteritic anterior ischemic optic neuropathy (NAION) and glaucoma^29,30^. There is definite different cause of disease – glaucomatous optic neuropathy starts with the compression of lamina cribrosa but NAION is caused by disorders of blood circulation in retrolaminar portion of the opbic nerve. However, the vascular incompetence could affect visual functional change or progression in glaucoma. So, NAION and NTG have common features in that retinal VD decreased compared with normal subjects^31^.

The present study has several limitations. First, there were small number of subjects with early glaucoma damage in this study. The further study with large number of patients are needed. Second, we used three machines to obtain RNFL thickness, macular segmentation thicknesses, and VDs. There could be issues of comparison between different machines. The absolute values of parameters may not be interchangeable between different machines, however, there are reports showing that correlation between different machines are good^32–34^. Therefore, analyzing correlations between the values from different machines could be performed. Third, there could be projection artifacts in en face imaging process. We had considered this interference when sorting out adequate subjects, however, it could hinder the precise evaluation of retinal capillary circulation. Fourth, although we were able to inspect only 3 × 3 mm macular scan when we conducted this study, it would have been more accurate if we used 6 × 6 mm scan which was known to have a higher diagnostic value^23^.

In spite of these limitations, in this study, it is remarkable that macular vascular density of deep layer may be an independent factor that affects central VF defect. Through OCTA, the effect of vascular incompetence can be visualized in deep layer of retina when we evaluate NTG patients with central scotoma. By considering both vascular circulation and thickness of macula, deterioration of central visual function in glaucoma patients could be predicted more precisely.

## Materials and Methods

### Study design and population

This cross-sectional study was performed according to the tenets of the Declaration of Helsinki. It was approved by the Institutional Review and Ethics Boards of Seoul St. Mary’s Hospital, South Korea. Written informed consent was obtained from all participants.

A total of 46 normal tension glaucoma (NTG) patients with central scotoma who attended Seoul St. Mary’s Hospital between March 2016 and January 2017 were enrolled in the study. The history of diabetes, hypertension or cardiovascular disease were documented and symptoms of vascular incompetence such as migraine, Raynaud’s phenomenon and hypotension were also recorded. Subjects with uncontrolled diabetes, uncontrolled hypertension or cardiovascular event with sequale were excluded.

The comprehensive ophthalmic examinations were done for all subjects including visual acuity, Goldmann applanation tonometry, slit-lamp examination, gonioscopy, automated perimetry using both 24-2 and 10-2 SITA program (Humphrey Visual Field Analyzer; Carl Zeiss Meditec, Inc, Dublin, CA, USA). Circumpapillary retinal nerve fiber layer (cpRNFL) and mGCIPL thickness were obtained using Cirrus spectral-domain OCT (Carl Zeiss Meditec, Inc, Dublin, CA). Macular structural segmentation was performed using Heidelberg SD-OCT device (Heidelberg Engineering, Heidelberg, Germany). OCT angiography was recorded by DRI OCT Triton system (Topcon, Tokyo, Japan).

All subjects included in this study should meet following criteria: (1) Best corrected visual acuity was 20/40 or better, (2) spherical equivalent(SE) was within ±5.0 diopters, (3) open angle on gonioscopy, and (4) intraocular pressure of lower than 21 mmHg. Exclusion criteria were as followings: (1) Patients with neurologic disease which could cause VF loss or retinal disease, (2) intracranial lesion which could make VF problem such as pituitary adenoma, (3) history of periorbital trauma, (4) advanced glaucomatous VF defect (mean deviation < −12dB) that may have diffuse central and peripheral VF loss.

### Definition of central visual field defect

A visual field test result was regarded as reliable when fixation loss was <20%, false-positive rate was <15%, and false-negative rate was <15%.

Initially to define subjects with central visual field defect (CVFD), we first analyzed SITA 24-2 results. CVFD was defined as VF defects within central 10° on pattern deviation probability map with clusters of three points with a probability of less than 5%, or two or more test points with a probability of less than 1% or smaller. All subjects had VF defects located within the superior or inferior hemifield of the central 10° regardless of the presence of defects outside the central 10° (Fig. 1).

Central retinal visual field function was evaluated through both SITA 24-2 and 10-2 results. Central retinal VF sensitivity was calculated by converting logarithmic dB scale to nonlogarithmic scale using formula [dB scale = 10 log(1/Lambert)] in central 12 points of SITA 24-2. Mean deviation (MD) and pattern standard deviation (PSD) were also evaluated in SITA 10-2.

### Macular vascular density by OCTA

OCTA scans were acquired by the DRI OCT Triton system (Topcon, Tokyo, Japan). The DRI OCT Triton system uses a swept source laser with a wavelength of 1050 nm and scan speed of 100,000 A-scans per second. The OCTA is based on Topcon OCT angiography ratio analysis (OCTARA) algorithm and 3 × 3 mm volume of macular scan was obtained. An active eye tracker was used to reduce motion artifact during imaging. The automated layer segmentation was performed for superficial vascular plexus (2.6 µm below internal limiting membrane to 15.6 µm below the junction between inner plexiform and inner nuclear layers (IPL/INL)) and deep vascular plexus (15.6 µm below IPL/INL to 70.2 µm below IPL/INL). En face projections of volumetric scans allow for visualization of structural and vascular details within segmented retinal layer boundaries^14,35^. Highly myopic or hyperopic eyes were excluded to minimize magnification or minification effect of en face images.

The images with image quality score over 70 were selected. Eyes with poor image qualities with following criteria were excluded: – (1) poor fixation resulting in double vessel pattern and motion artifacts, (2) blurred image that hinder the clarity of vessel contour, and (3) macular segmentation error. The quality of each image was independently evaluated by two glaucoma specialists (SJJ and HYP).

**Figure 5:**
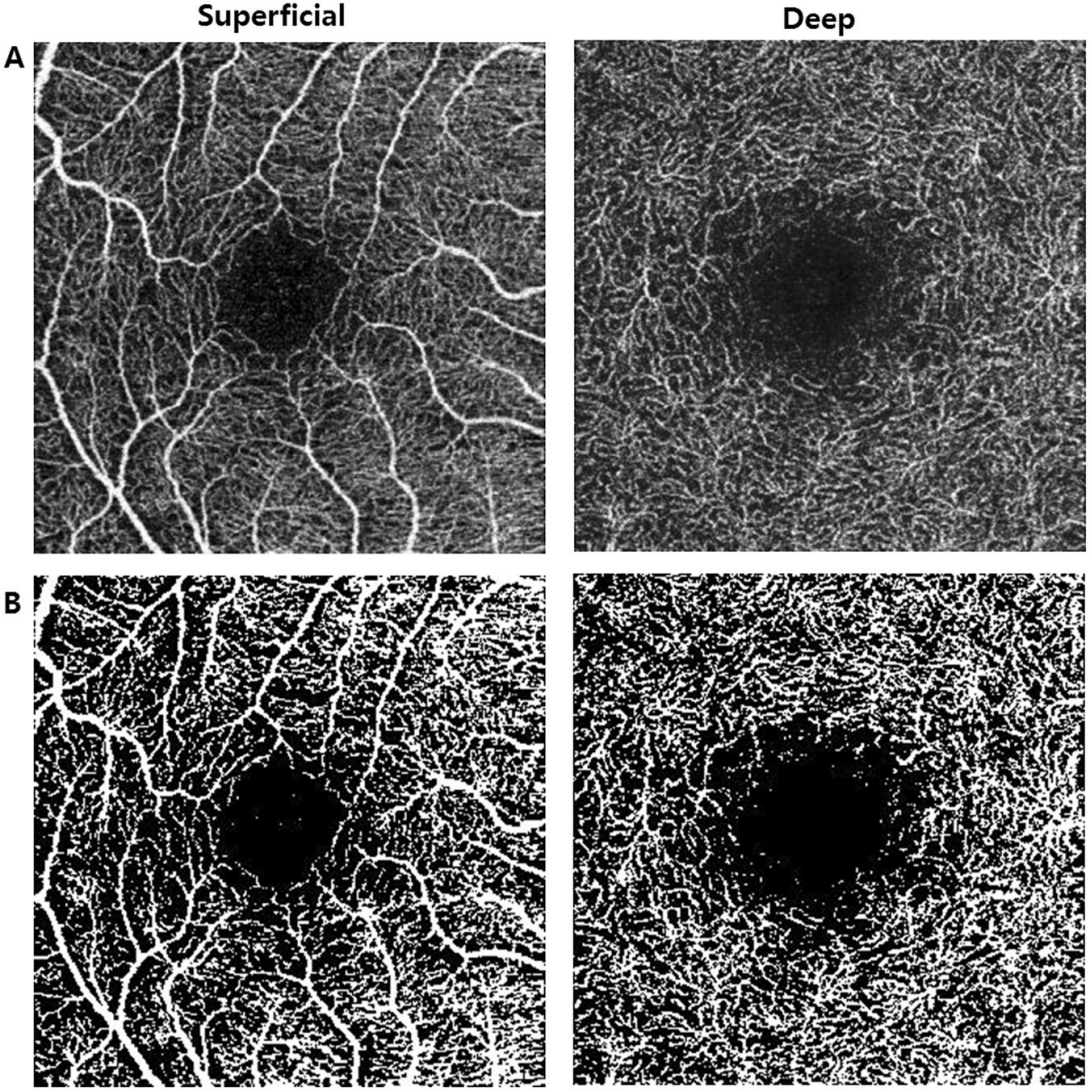
(**A**) OCTA en face image of superficial and deep retinal vascular layer. (**B**) Binarized image of en face OCTA results using thresholding algorithm by ImageJ Software. The white area was considered to be vascular lumen and calculated as percentage of total area.

To calculate macular vascular density (VD), ImageJ software (National Institutes of Health, Bethesda, MD, USA) was used. As shown in other studies, a binary slab was created according to the ImageJ ‘mean threshold’ algorithm, which automatically computes the threshold value as the mean of the local grayscale distribution. Each binarized 8-bit image was converted into red-green-blue (RGB) color model and then split into the three channels (red, green, and blue). After assigning white pixels as vessel and black pixels as background, vascular density was defined as the ratio between vessel pixels and the total area (Fig. 5)^16,36,37^. Intraobserver repeatability of our VD measurement was also calculated from data of 20 normal eyes tested twice.

### Measurement of segmented macular thickness

All patients underwent macular structural segmentation using Spectralis SD-OCT device (Heidelberg Engineering, Heidelberg, Germany). OCT scans were performed by the same experienced operator. The OCT scan images of included patients were absence of movement artifact and well centered.

Automated macular segmentation was performed by stored software – assigning retinal boundaries of the inner limiting membrane(ILM), the boundaries between the RNFL and the ganglion cell layer (GCL), the GCL and the IPL, the IPL and the INL, the INL and outer plexiform layer (OPL), the OPL and the outer nuclear layer (ONL). To minimize segmentation errors, segmented layer was manually verified and performed repeatedly.

**Figure 6:**
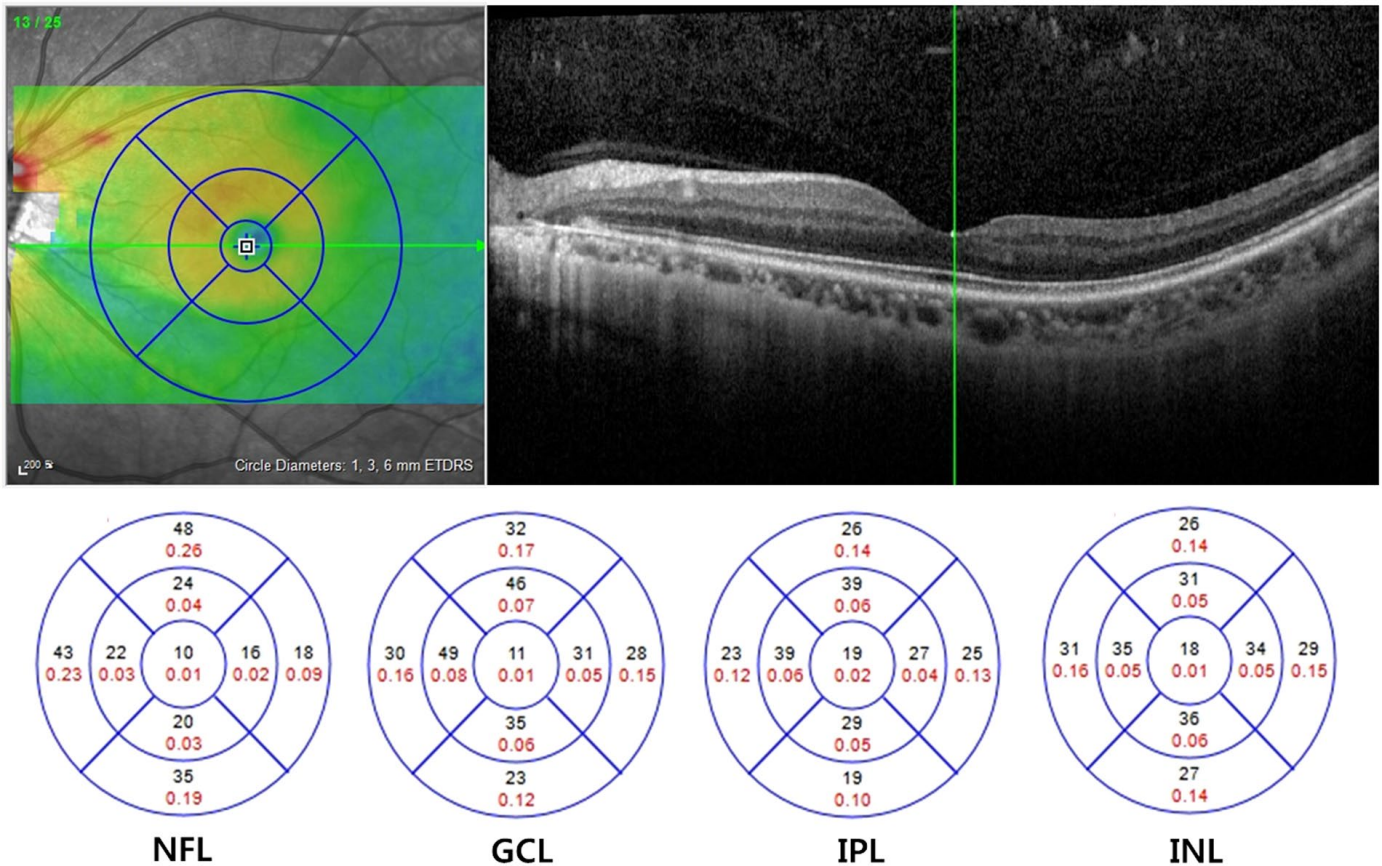
Macular structural segmentation image using Spectralis SD-OCT. The segmented retinal thickness map shows three concentric rings with diameters of 1, 3, and 6 mm. The average thickness of each segmented layer was calculated as the average value of 8 subfields that excluded central foveola.

The segmented retinal thickness map shows three concentric rings with diameters of 1, 3, and 6 mm. The intermediate and outer rings were divided into quadrants by two intersecting lines and the thickness of each zone was separately measured as follows: inner superior, inner nasal, inner inferior, inner temporal, outer superior, outer nasal, outer inferior, and outer temporal zone. The average thickness of each segmented layer was calculated as the mean value of 8 subfields excluding central foveola area with a 1-mm radius (Fig. 6)^38^. The volume of each layer within a 6-mm diameter was also measured automatically.

### Statistical analysis

All statistical analyses were performed with SPSS version 24.0 (SPSS Inc., Chicago, IL). P < 0.05 was considered statistically significant. Descriptive results were calculated as the value of mean and standard deviation. The Student t-tests was used to evaluate structural and perfusional differences between groups divided according to the severity of central VF defect. The Shapiro-Wilk analysis was used for assessing normality and Pearson correlation analysis was used to evaluate the relationships between thickness of cpRNFL, thickness of GCIPL, thicknesses and volumes of segmented macular layers (NFL, GCL, IPL, INL, OPL and ONL), functional parameters of perimetry and macular vascular densities. Univariate and multivariate linear regression analyses were performed to identify significant factors that affected the functional values of VF tests. The logarithmic regression line was used to divide subjects into better or worse functional group than expected from the macular thickness. We performed VD comparisons between those groups using Student t-test. The statistical significance of differences in R square values of different regression analysis models was also assessed by multivariate analysis of variance (MANOVA).

## Electronic supplementary material

Supplementary table
