# Supplementary material for: Effect of Macular Vascular Density on Central Visual Function and Macular Structure in Glaucoma Patients
Source: Sci Rep. 2018 Oct 30;8:16009. doi: 10.1038/s41598-018-34417-4 (PMC6207782; doi:10.1038/s41598-018-34417-4)
Supplement: Supplementary file 1 — Supplementary table [file 41598_2018_34417_MOESM1_ESM.docx]

**Effect of Macular Vascular Density on Central Visual Function and Macular Structure in Glaucoma Patients**

*Soo Ji Jeon, Hae-Young Lopilly Park, Chan Kee Park*

Department of Ophthalmology, Seoul St. Mary’s Hospital, College of Medicine,

The Catholic University of Korea, Seoul, Republic of Korea

**Corresponding author**

Chan Kee Park, MD, PhD

Department of Ophthalmology, Seoul St. Mary’s Hospital, College of Medicine, The Catholic University of Korea, 222, Banpo-daero, Seocho-gu, Seoul, 06591, Republic of Korea

E-mail: [ckpark@catholic.ac.kr](mailto:ckpark@catholic.ac.kr)

Tel : 82-2-2258-1188

Fax : 82-2-599-7405

Table 1. Univariate and multivariate regression analysis of MD of SITA 10-2

|  | Univariate analysis | | Multivariate analysis  Model 1 | | Multivariate analysis  Model 2 | |
| --- | --- | --- | --- | --- | --- | --- |
|  | β ± SE | *P* value | β ± SE | *P* value | β ± SE | *P* value |
| Superficial VD* | 0.421 ± 0.225 | 0.068 | 0.109 ± 0.210 | 0.608 |  |  |
| Deep VD | 1.253 ± 0.446 | 0.007 | 0.676 ± 0.427 | 0.121 | 0.851 ± 0.409 | 0.044 |
| NFL average thickness | 0.612 ± 0.215 | 0.006 | -0.323 ± 0.392 | 0.415 |  |  |
| GCL average thickness | 0.533 ± 0.126 | <0.001 | 1.039 ± 0.386 | 0.010 | 0.461 ± 0.126 | 0.001 |
| IPL average thickness | 0.687 ± 0.229 | 0.004 | -0.747 ± 0.521 | 0.159 |  |  |
| INL average thickness | -0.488 ± 0.307 | 0.119 |  |  |  |  |
| OPL average thickness | -0.445 ± 0.224 | 0.843 |  |  |  |  |
| ONL average thickness | -0.082 ± 0.110 | 0.461 |  |  |  |  |

* Superficial VD excluding large vessels

Model 1 included the factors of p value lower than 0.1 in univariate analysis.

Modes 2 used the backward elimination method.

cpRNFL = Circumpapillary retinal nerve fiber layer; mGCIPL = Macular ganglion cell-inner plexiform layer; VD = Vascular density; NFL = Nerve fiber layer; GCL = Ganglion cell layer; IPL = Inner plexiform layer; INL = Inner nuclear layer, OPL = Outer plexiform layer; ONL = Outer nuclear layer

Table 2. Univariate and multivariate regression analysis of unlogged center sensitivity of SITA 24-2

|  | Univariate analysis | | Multivariate analysis  Model 1 | | Multivariate analysis  Model 2 | |
| --- | --- | --- | --- | --- | --- | --- |
|  | β ± SE | *P* value | β ± SE | *P* value | β ± SE | *P* value |
| Superficial VD* | 41.652 ± 18.994 | 0.034 | 25.872 ± 19.443 | 0.191 |  |  |
| Deep VD | 105.595 ± 38.201 | 0.008 | 68.789 ± 39.240 | 0.087 | 85.636 ± 38.491 | 0.031 |
| NFL average thickness | 28.791 ± 19.376 | 0.144 |  |  |  |  |
| GCL average thickness | 30.001 ± 11.938 | 0.016 | 54.122 ± 28.886 | 0.068 | 22.896 ±.11.874 | 0.060 |
| IPL average thickness | 35.277 ± 20.795 | 0.097 | -65.110 ± 48.322 | 0.185 |  |  |
| INL average thickness | -73.836 ± 24.495 | 0.103 |  |  |  |  |
| OPL average thickness | -16.944 ± 18.982 | 0.377 |  |  |  |  |
| ONL average thickness | -11.326 ± 9.333 | 0.231 |  |  |  |  |

* Superficial VD excluding large vessels

Model 1 included the factors of p value lower than 0.1 in univariate analysis.

Modes 2 used the backward elimination method.

cpRNFL = Circumpapillary retinal nerve fiber layer; mGCIPL = Macular ganglion cell-inner plexiform layer; VD = Vascular density; NFL = Nerve fiber layer; GCL = Ganglion cell layer; IPL = Inner plexiform layer; INL = Inner nuclear layer, OPL = Outer plexiform layer; ONL = Outer nuclear layer
